# Prokineticin 2 regulates the electrophysiological activity of gonadotropin-releasing hormone neurons via direct signalling in adult female mice

**DOI:** 10.3389/fendo.2026.1809385

**Published:** 2026-05-20

**Authors:** Imre Farkas, Barbara Göblyös, Davide Moi, Valentina Onnis, Imre Kalló, Zsolt Liposits

**Affiliations:** 1 Laboratory of Reproductive Neurobiology, HUN-REN Institute of Experimental Medicine, Budapest, Hungary; 2 Roska Tamás Doctoral School of Sciences and Technology, Faculty of Information Technology and Bionics, Pázmány Péter Catholic University, Budapest, Hungary; 3 Department of Life and Environmental Sciences, Unit of Pharmaceutical, Pharmacological and Nutraceutical Sciences, University of Cagliari, Cittadella Universitaria di Monserrato, Monserrato, Italy

**Keywords:** confocal microscopy, female mouse, GnRH neuron, *in situ* hybridization, patch clamp, prokineticin 2, RNAscope, slice electrophysiology

## Abstract

**Introduction:**

Prokineticin 2 (PK2) signaling to gonadotropin-releasing hormone (GnRH) neurons is essential for their embryonic migration from the nasal placode to the brain, and its disruption results in hypogonadotropic hypogonadism. Although PK2 has been implicated in the regulation of reproductive function in adult rodents, its direct cellular targets remain insufficiently defined. Here, we investigated whether GnRH neurons in adult female mice receive and functionally respond to PK2 signals.

**Methods:**

Whole-cell patch-clamp recordings were obtained from acute forebrain slices prepared from adult female GnRH-GFP mice. The effects of PK2 and prokineticin receptor antagonists on spontaneous firing and miniature postsynaptic currents (mPSCs) were assessed. Expression of PK2 receptor (PKR2) in GnRH neurons was examined using RNAscope *in situ* hybridization for simultaneous detection of PKR2 and GnRH mRNAs, followed by confocal laser microscopic analysis.

**Results:**

PK2 increased action potential firing and mPSC frequency in 42.9% (firing) and 42.1% (mPSC) of GnRH neurons. These effects were prevented by intracellular G-protein inhibition with GDP-β-S and by pharmacological blockade of prokineticin receptors using PKRA7 or PC27. Inhibition of nitric oxide synthase by NPLA also eliminated the PK2-induced elevation of mPSC frequency. RNAscope analysis revealed PKR2 mRNA expression in approximately one-third of GnRH neurons.

**Discussion:**

These findings demonstrate that PK2 directly enhances the excitability of a subset of GnRH neurons through PKR2-dependent mechanisms, identifying these neurons as functional targets of PK2 signaling in the mature female mouse brain.

**Conclusion:**

PK2 serves as a direct modulator of the reproductive neuroendocrine axis in adult female mice by activating PKR2 expressed in a subpopulation of GnRH neurons.

## Introduction

Prokineticin 1 (PK1) and prokineticin 2 (PK2) are secreted, biologically active peptides that influence the function of several organ systems in mammals, including the operation of the nervous system (1, 2). Both prokineticins are synthesized in the brain, although, with different topography. PK1 is synthesized exclusively in the medulla oblongata by neurons residing in the nucleus of the solitary tract and in the vicinity of the reticular nucleus, while PK2 is principally produced in multiple loci of the forebrain (3, 4). Regarding the distribution of their cognate receptors, prokineticin receptor 2 (PKR2) is more widely and robustly expressed in the neuroaxis (3, 5) compared to allocation of prokineticin receptor 1 (PKR1). It is of note, that the amino acid sequence of the two prokineticin receptors is highly conserved and they show an 85% homology (6–8).

The central regulation of reproduction is governed by gonadotropin-releasing hormone (GnRH) (9) produced by forebrain neurons in rodents (10–14). A peculiar feature of the GnRH system is that its composing neurons derive from the olfactory placode, invade the olfactory bulb and migrate along the path of the terminal nerve toward their final destination in the basal forebrain (15–18). Failure of migration of neuronal precursors of olfactory and GnRH cells results in Kallmann syndrome (KS), a congenital form of hypogonadotropic hypogonadism associated with partial or total anosmia in humans (19). The PK2/PKR2 signalling pathway has been identified as a main regulator of olfactory bulb development and migration of GnRH neurons since *PK2* and *PKR2* knock-out (KO) mice mimicked the symptoms of KS (20, 21). As far as the reproductive neuropathology part of the KO phenotypes is concerned, the majority of GnRH neurons was arrested in their migration at the cribriform plate, accordingly only a small fraction of GnRH neurons reached their final residence in the preoptic area and the hypophysiotropic GnRH axon projections toward the release sites of the neurohormone in the median eminence have almost totally failed (21, 22). The loss of the trophic effects of the hypothalamo-pituitary unit upon the gonads resulted in halted gametogenesis in both sexes (21, 22). The regulatory role of the prokineticin 2 signalling in human reproduction has also been substantiated (22–24), although several pieces of this complex puzzle still await clarification (25).

Contrasting the pivotal role of PK2 in orchestrating the migration and proper formation of the GnRH neuron assembly in the medial septum (MS) - diagonal band of Broca (DBB) - medial preoptic area (MPOA) continuum of the mouse brain, our contemporary scientific knowledge about the impact of this neuropeptide in the central control of reproduction in adult rodents is rather limited. It has previously been shown that the estrus cycle is dependent on PK2 signalling (26). In PK2-null and PKR2 null compound heterozygous mice, the estrus cycle was prolonged and showed irregularities. In proestrous mice, administration of the PKR2 antagonist, 3Cl-MPL, blocked the estrus cycle and blunted the circulating luteinizing hormone (LH) levels. PK2 also influences the hypothalamo-pituitary-gonadal axis in males. A recent study has demonstrated (27) that chronic intracerebroventricular infusion of PK2 at nanomolar concentration increased the expression of GnRH mRNA, augmented the secretion of follicle-stimulating hormone (FSH), luteinizing hormone (LH) and testosterone and increased the seminiferous epithelial thickness in the testis.

A conceivable explanation for the effects would be a direct targeting of GnRH neurons by PK2. Despite of this plausible idea, demonstration of presence of PKR2 in GnRH neurons has not been successful, so far. Studies by Pitteloud and colleagues (22) for example have thoroughly examined this possibility by simultaneous detection a PKR2-mRNA expressing sites and GnRH-immunoreactive neurons in mice. Neither developing (E13.5) nor mature, adult GnRH neurons were found to express PKR2-mRNA revealed by conventional *in situ* hybridization histochemistry (ISHH). Engineering a PKR2-Cre mouse line with green fluorescent protein (GFP) reporter gene (5) allowed the mapping of PK2R-expressing loci in the brain. This approach has confirmed the wide distribution of the receptor within the brain and its absence from GnRH neurons. Furthermore, the comparison of female and male brains revealed a marked sexual dimorphism in the expression of PKR2-Cre. In females, higher Cre activity was observed in neurons of the MPOA, ventromedial nucleus (VMN) and arcuate nucleus (ARC), the acknowledged neural regulatory centers of reproduction.

To resolve the discrepancy between the hypothetical yet plausible direct targeting of GnRH neurons by PK2 and the reported absence of its receptor in these neurons, the present study aimed to elucidate the role of PK2 in the direct regulation of GnRH neurons using methodologies that are more sensitive than those applied in previous studies. Furthermore, since retrograde endocannabinoid (28, 29) and nitric oxide (30, 31) signalling are specialized mechanisms of neural communication where signals are produced by the postsynaptic neuron and travel “backwards” to activate receptors on the presynaptic axon terminal and GnRH neurons possess both retrograde machineries (32, 33) involvement of these mechanisms was also examined.

Patch clamp electrophysiology and highly sensitive RNAscope technique were applied to clarify the cellular target/s of the hormone in adult, female mice.

## Materials and methods

### Animals

Adult, female CD1 (n = 14) and GnRH-GFP mice (34) (n=40) were used (age: 112 ± 32 day). The animals were obtained from local colonies bred at the Medical Gene Technology Unit of the HUN-REN Institute of Experimental Medicine. They were housed under controlled lighting (12:12 h light–dark cycle, lights on at 07:00 h) and temperature (22 ± 2 °C) conditions, with access to food (Ssniff S8189-S095) and water *ad libitum*.

The gonadal cycle stage of the experimental animals was not monitored.

### Slice electrophysiology

Brain slice preparation was carried out as described earlier (33). Briefly, the brains of adult female GnRH-GFP mice were removed from the skull under deep isoflurane anaesthesia, then immersed in ice-cold, low-Na cutting solution continuously bubbled with carbogen, a mixture of 95% O_2_ and 5% CO_2_. The cutting solution contained the following (in mM): sucrose 205, KCl 2.5, NaHCO_3_ 26, MgCl_2_ 5, NaH_2_PO_4_ 1.25, CaCl_2_ 1, and glucose 10. Hypothalamic blocks were dissected, and 220-μm-thick coronal slices were prepared from the mPOA with a VT-1000S vibratome (Leica Microsystems, Wetzlar, Germany) in the ice-cold, low-Na, oxygenated cutting solution. The slices containing preoptic area (POA) were transferred into artificial cerebrospinal fluid (aCSF) (in mM): NaCl 130, KCl 3.5, NaHCO_3_ 26, MgSO_4_ 1.2, NaH_2_PO_4_ 1.25, CaCl_2_ 2.5, and glucose 10, bubbled with carbogen, and left for 1 h to equilibrate. Equilibration started at 33°C, and it was allowed to cool down to room temperature.

Recordings were carried out in carbogenated aCSF at 33°C. Axopatch-200B patch-clamp amplifier, Digidata-1550B data acquisition system, and pCLAMP 10.7 software (Molecular Devices Co., Silicon Valley, CA, USA) were used for recording. Neurons were visualized with a BX51WI IR-DIC microscope (Olympus Co., Tokyo, Japan). The patch electrodes (OD = 1.5 mm, thin wall; WPI, Worcester, MA, USA) were pulled with a Flaming-Brown P-97 puller (Sutter Instrument Co., Novato, CA, USA).

GnRH-GFP neurons were identified by brief illumination at 470 nm (CoolLED pE-100, Andover, UK) using an epifluorescent filter set based on their green fluorescence, typical fusiform shape, and characteristic topography (34) in the diagonal band of Broca, the organum vasculosum of the lamina terminalis (OVLT), and the medial preoptic area (mPOA).

### Whole-cell patch clamp recording

Whole-cell patch-clamp measurements started with a control recording (1 min for firing recordings and 2 mins for mPSC recordings), then prokineticin 2 (PK2) was pipetted into the aCSF-filled measurement chamber containing the brain slice in a single bolus, and the recording continued for further 8 mins for firing recordings and 9 mins for mPSC recordings. Flow rate in the chamber was 3 ml/min, and the chamber volume was 1.5 ml. Prokineticin 2 was applied as 10 µM stock solution, of which 4 µl was added to the chamber. Under these circumstances the final concentration of prokineticin 2 was 26 nM (i.e., 4 µl of 10 µM solution diluted in 1.5 ml). Given that both aCSF and prokineticin 2 are highly diluted, their mixing can be considered effectively instantaneous. Therefore, the final concentration reaching the measured GnRH neuron can be assumed to be 26 nM. Pre-treatment with the extracellularly applied antagonists started 10 minutes before starting the recording, and the antagonist was continuously present in the aCSF during the electrophysiological recording. Each neuron served as its own control when drug effects were evaluated.

The miniature post-synaptic currents (mPSCs) in GnRH neurons were measured as described earlier (33). Briefly, the neurons were voltage clamped at −70 mV of holding potential. The intracellular pipette solution contained the following (in mM): HEPES 10, KCl 140, EGTA 5, CaCl_2_ 0.1, Mg-ATP 4, and Na-GTP 0.4 (pH = 7.3 with NaOH). The resistance of the patch electrodes was 2–3 MΩ. Only cells with a low holding current (10 pA) and a stable baseline were used. Input resistance (R_in_), series resistance (R_s_), and membrane capacitance (C_m_) were also measured before and after each treatment by using 5 mV hyperpolarizing pulses. To ensure consistent recording qualities, only cells with R_s_ <20 MΩ, R_in_ >500 MΩ, and C_m_ >10 pF were accepted. Spike-mediated transmitter release was blocked in all mPSC experiments by adding the voltage-sensitive Na-channel inhibitor tetrodotoxin (TTX; 660 nM, Tocris) to the aCSF 10 min before mPSCs were recorded. The control period spanned 0–2 min, followed by the PK2-treated period from 2–10 min, yielding a total recording duration (control+treated) of 10-min-long.

Action potentials were recorded in whole-cell current clamp mode at 0 pA. The control period spanned 0–1 min, followed by the PK2-treated period from 1–9 min, resulting in a total recording duration (control+treated) of 9 min. The antagonist pretreatment started 10 minutes before starting the recording. Each neuron served as its own control when drug effects were evaluated.

G-protein coupled receptors (GPCRs) including PK2-receptors were blocked intracellularly in the recorded neurons by adding the membrane-impermeable guanosine 5′-[β-thio]diphosphate trilithium salt (GDP-β-S, 2 mM, Sigma) to the intracellular (pipette) solution. After achieving the whole-cell configuration, measurements started after 15 minutes of equilibration to reach a stable intracellular milieu.

### Chemicals used for electrophysiology

Tetrodotoxin (TTX, 660 nM, Tocris No.1078) (35).

GDP-β-S (2 mM, Sigma G7637) (32).

Prokineticin 2 (26 nM, Sigma SRP3146) (36).

PKRA7 (prokineticin 1 and 2 receptor inhibitor, 2 µM, Tocris No.6238) (37).

PC27 (selective prokineticin 2 receptor inhibitor, 1 µM, source: Valentina Onnis, University of Cagliari) (38).

AM251 (cannabinoid receptor type-1 [CB1] inverse agonist, 1 µM, Tocris No.1147) (32).

NPLA (neuronal NO-synthase [nNOS] inhibitor, 1 µM, Tocris No.1200) (32).

### Statistical analysis used for electrophysiology

Recordings were stored and analysed offline. Event detection was performed using the Clampfit module of the PClamp 10.4 software (Molecular Devices Co., Silicon Valley, CA, USA). Firing rates and mPSC frequencies were calculated as the number of APs or mPSCs divided by the length of the corresponding time. The mean values of the control and treated periods of the recording were calculated from these frequency values. Group data were expressed as mean ± standard error of mean (SEM). Two-tailed Student’s t-test, or Wilcoxon test was applied for comparison of groups. Cohen’s *d*-test, Welch’s two-sample t-test, Shapiro-Wilk test and non-parametric Wilcoxon test were performed to ensure the robustness of sample sizes of those measurements involving n ≤ 10 recordings. Differences were considered significant at p < 0.05.

Frequency distribution curves of recordings were generated using a 30-second-wide sliding window applied across the full duration of each recording. Frequencies were calculated within each window, with the window advanced by 5-second increments from the beginning to the end of the recording.

### Clustering of firing and mPSC data

The following analysis was performed separately for mPSC (n = 19) and firing data (n = 21). For each cell, frequency responses were normalized to baseline (100%) to quantify the relative change following PK2 application.

Based on the observed response patterns, the data suggested the presence of two subgroups: a PK2-sensitive group showing marked changes from baseline and a non-responsive group showing minimal changes. To avoid applying a subjective threshold for subgroup classification, Euclidean distances between samples were calculated, and hierarchical clustering was performed using Ward’s method, which minimizes within-cluster variance. The resulting dendrogram indicated a potential separation into two subgroups at the first major branch point.

Based on this data-driven subdivision, cells were assigned to responder-like and non-responder-like clusters. Differences between these two clusters were assessed using Mann–Whitney U-tests.

All statistical analyses were performed using R Studio (Version 2024.12.0 + 467) and Statistica (version 14.2.0).

### RNAscope *in situ* hybridization

Animals (n = 6) were perfused under deep anesthesia (ketamine 25 mg/kg b.w. and xylazine 5 mg/kg b.w.) with 4% PFA solution made and stored in RNase-free PBS (0.1M, pH 7.4) at 4 °C until use. The brains were removed, and 25 μm-thick coronal sections were cut on a Leica VT 1000S Vibratome (Leica Microsystems) from a block spanning Bregma levels from +1.21 mm to –2.69 mm. Sections were collected at a consecutive manner into four different wells containing RNase-free PBS, thus each section pool contained every fifth sections being 100 µm apart. Then sections covering the region of GnRH occurrence were selected from a single pool, washed with RNase-free PBS (3x20 min), pre-treated with 0,3% H_2_O_2_ (20 min), mounted onto Superfrost Ultra Plus slides, air-dried, baked at 60 °C (1 h) and finally boxed for storage at –20 °C. Prior to hybridization, boxes were equilibrated to room temperature to avoid humid precipitation. Sections were then processed according to the manufacturer’s protocol (RNAscope^®^ Multiplex Fluorescent Reagent Kit v2, Document Number 323100-USM) with some modifications introduced by Kormos et al. (39) for optimizing concurrent mRNA and protein detection in 30 μm paraformaldehyde-fixed sections. Tissue quality was tested by employing positive and negative control probes (catalogue #320881, #313911 and #310043, respectively) on test slides of the brains.

### Probes used for RNAscope ISH

Differently tagged target probes were used in the hybridization cocktails. For sections of CD1 mice the channel 1 probe was Mm-GnRH1-O1 (Mus musculus gonadotropin releasing hormone 1; #476281) and the channel 2 probe was Mm-Prokr2-C2 (Mus musculus prokineticin receptor 2 (Prokr2) mRNA; #498431-C2). For sections of GnRH-GFP mice the Mm-GnRH1-O1-C3 (#476281-C3) was combined with Mm-Prokr2-C2 (#498431-C2). For signal visualization, Opal 520, Opal 570 and Opal 690 dyes (1:1000) were used. Finally, slides were coverslipped with Prolong Antifade kit (Molecular Probes, Leiden, The Netherlands) following a short counterstaining of sections with DAPI.

### Capturing and analysing the RNAscope signals

The double-labelled sections were scanned by a slide scanner (Pannoramic Scan; 3DHISTECH) using a 20× objective magnification. Selected regions of the sections containing GnRH neurons underwent confocal analyses using a Nikon A1R confocal microscope (Nikon, Japan) at 20× and 60× objective magnification. Laser intensities and other acquisition parameters were kept the same during the whole scanning. Multiple stacks of optical slices (1024 × 1024 pixels, z-steps 0.3-0.5 µm) were obtained to enable 3D reconstruction of the perikaryon and the proximal dendrites of GnRH neurons. Co-localization of PKR2 mRNA and GnRH mRNA signals was validated in orthogonal views of the fluorescent labels, confirming their position within the cellular border. The mean number of PKR2 mRNA expressing GnRH neurons was determined by localizing the PKR2 mRNA signals in phenotypically identified and randomly selected GnRH neurons (~40–80 neurons/brain) from medial septum and medial preoptic area of each brain. Channel intensity optimization and pseudocoloring were performed in the ImageJ image analysis software (40).

Information about the used probes and dyes is shown in **Supplementary Table 1**.

## Results

### PK2 increases the firing rate of GnRH neurons

**Figure 1:**
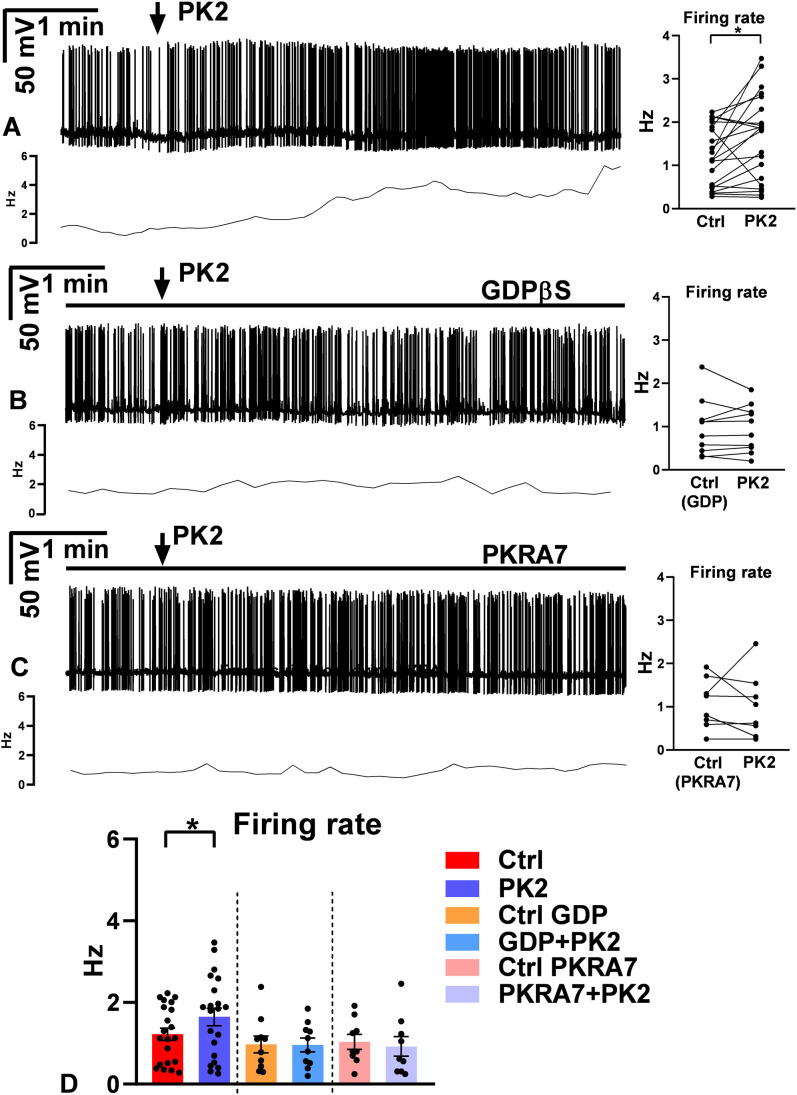
Firing rate responses of GnRH neurons upon prokineticin 2 administration. **(A)** Prokineticin 2 (PK2) application (arrow) significantly increased the firing activity of GnRH neurons (Wilcoxon paired test, n=21, p=0.0117). **(B)** Intracellularly applied G-protein blocker, GDP-β-S (GDP), eliminated the effect PK2 (Student’s t-test, n=10, p=0.8422). **(C)** The prokineticin receptor antagonist, PKRA7 also prevented the action of PK2 (Student’s t-test, n=9, p=0.573). **(D)** Bar graph confirms a significant facilitatory effect of PK2. Horizontal lines show presence of inhibitors. Frequency distribution curve below each recording shows changes in the frequency.

**Figure 2:**
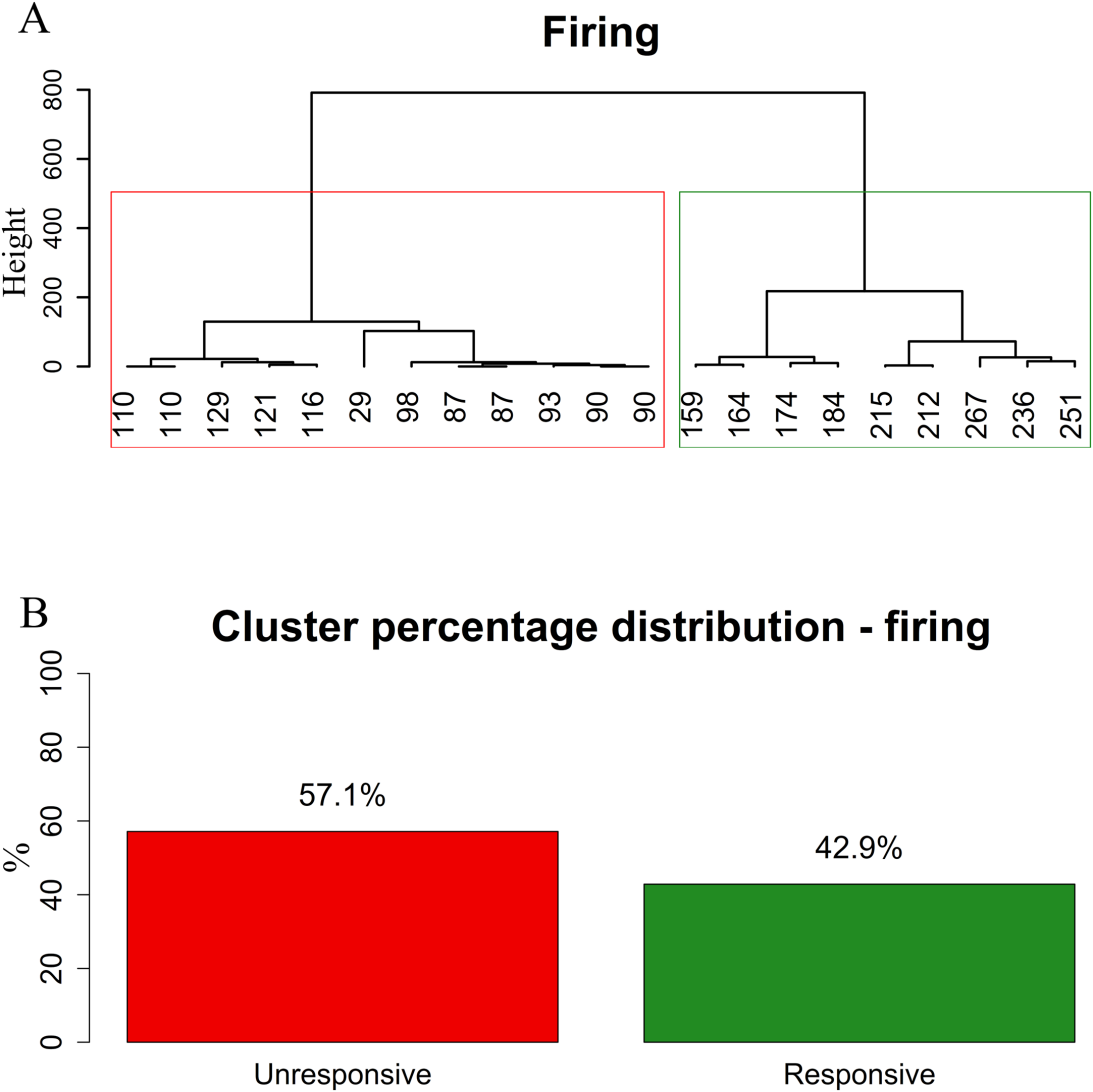
Clustering of firing data reveals two distinct GnRH neuron populations based on responsiveness to PK2. Euclidean distance was calculated among samples, and clusters were generated using the Ward’s method which minimizes total within-cluster variance. The dendrograms show a clear separation at the first major branch point in the firing datasets which was used to define two subgroups. Mann–Whitney U-tests were performed to compare the two clusters. Significant difference was found in firing parameters (*p* < 0.001). **(A)** Hierarchical clustering of firing data using Ward’s method. **(B)** Proportion of PK2-responsive and unresponsive GnRH neurons in the two firing-based clusters. Values below the mathematical graph tree-like representation are percentage changes in the firing rate upon PK2 administration.

To evaluate the effect of PK2 on the electric activity of GnRH neurons, firing was recorded in whole-cell current clamp mode at 0 pA holding current. After the control period (1 min), a single bolus of PK2 (26 nM) elevated the firing rate significantly (ctrl: 1.2 ± 0.15 Hz, PK2: 1.6 ± 0.22 Hz, Wilcoxon test, p=0.0117, n=21) (**Figures 1A, D**; **Supplementary Table 2**). In addition, a more detailed analysis uncovered that the population of GnRH neurons is inhomogeneous considering their responsivity to PK2 application. Cluster analysis clearly identified an unresponsive (firing rate: ctrl: 1.4 ± 0.23 Hz, PK2: 1.3 ± 0.26 Hz, n=12) and a responsive (firing rate: ctrl: 1.0 ± 0.16 Hz, PK2: 2.1 ± 0.33 Hz, n=9) group of GnRH neurons (Mann-Whitney U-test, p=0.001) (**Figure 2**, **Supplementary Table 2**). To shed light on whether this effect was direct on the measured GnRH neuron, the G-protein coupled receptors were blocked intracellularly by pre-treating the cell with the membrane-impermeable GDP-β-S (2 mM). This pretreatment prevented the action of PK2 (GDP-ctrl: 0.98 ± 0.20 Hz, PK2: 0.96 ± 0.17 Hz, Student’s t-test, t= 0.2049, df=9, p= 0.8422, n=10) (**Figures 1B, D**; **Supplementary Tables 2**, **3**). Further measurements revealed that PK2 acted via prokineticin receptors because antagonizing them by the prokineticin receptor antagonist PKRA7 (2 µM) pretreatment also eliminated the PK2-evoked elevation of the firing rate (PKRA7-ctrl: 1.0 ± 0.18 Hz, PK2: 0.93 ± 0.24 Hz, Student’s t-test, t= 0.5876, df=8, p= 0.573, n=9) (**Figures 1C, D**; **Supplementary Tables 2**, **3**).

### PK2 enhances frequency of miniature postsynaptic currents in GnRH neurons

**Figure 3:**
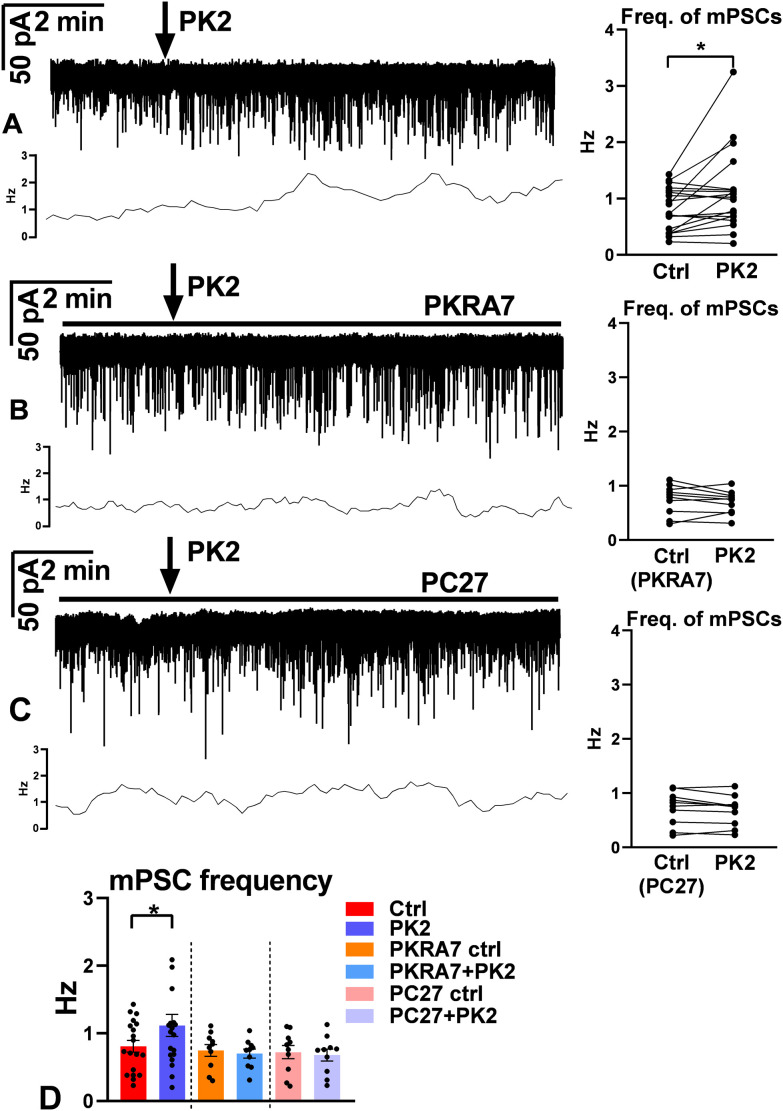
mPSC responses of GnRH neurons upon PK2 administration in tetrodotoxin (TTX) exposed slices. **(A)** PK2 application (arrow) significantly elevated the frequency of mPSCs in GnRH neurons (Wilcoxon paired test, n=19, p=0.0411). **(B, C)** Both prokineticin receptor antagonists, PKRA7 and PC27, prevented the action of PK2 (Student’s t-test, PKRA7: n=10, p=0.3145; PC27: n=10, p=0.1275). **(D)** Bar graph confirms a significant facilitatory effect of PK2. Horizontal lines show presence of inhibitors. Frequency distribution curve below each recording shows changes in the frequency.

**Figure 4:**
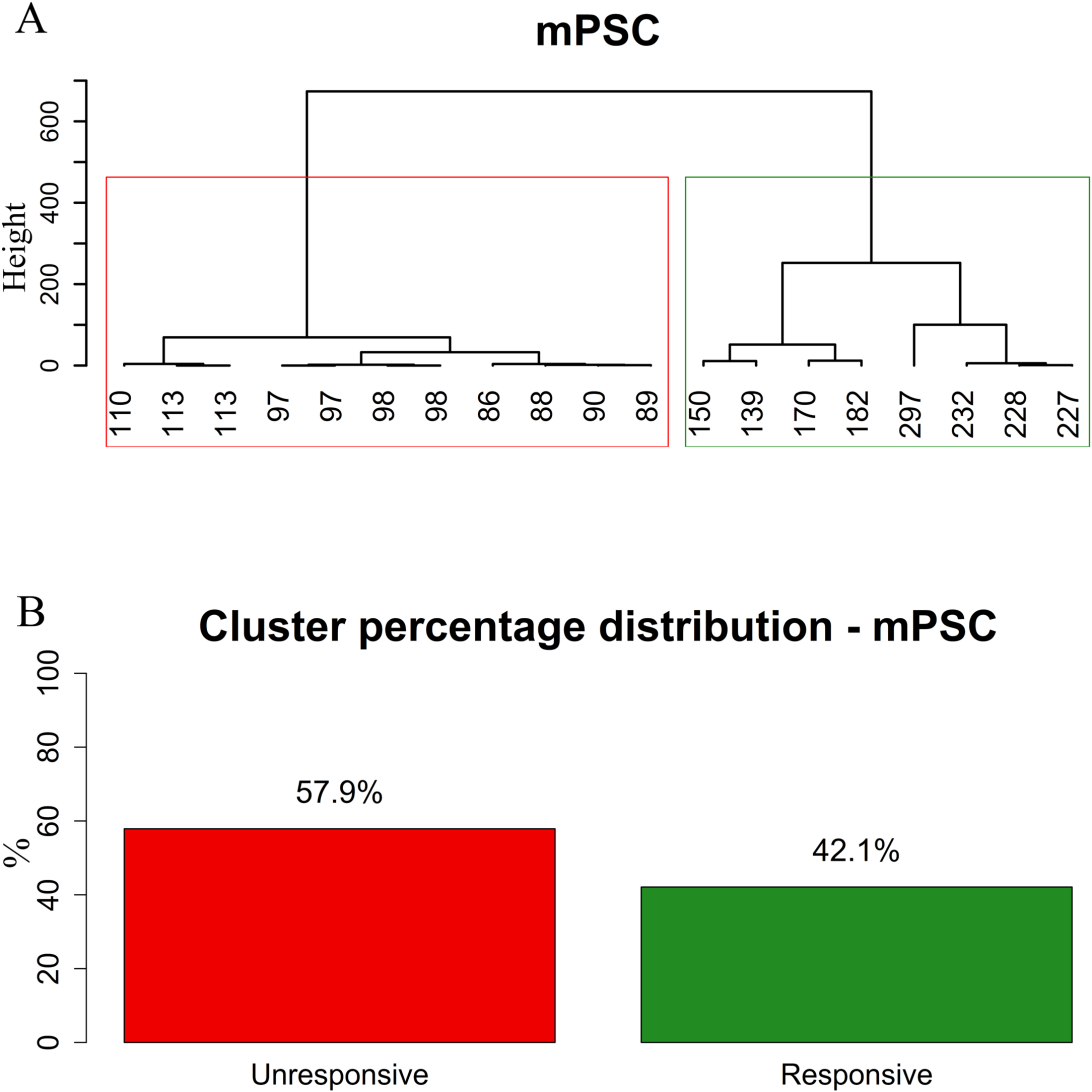
Clustering of mPSC data reveals two distinct GnRH neuron populations based on responsiveness to PK2. Euclidean distance was calculated among samples, and clusters were generated using the Ward’s method which minimizes total within-cluster variance. The dendrograms show a clear separation at the first major branch point in the mPSC datasets which was used to define two subgroups. Mann–Whitney U-tests were performed to compare the two clusters. Significant difference was found in mPSC parameters (*p* < 0.001). **(A)** Hierarchical clustering of mPSC data using Ward’s method. **(B)** Proportion of PK2-responsive and unresponsive GnRH neurons in the two mPSC-based clusters. Values below the mathematical graph tree-like representation are percentage changes in the frequency of mPSCs upon PK2 administration.

Single bolus of PK2 increased the frequency of mPSCs significantly (ctrl: 0.81 ± 0.087 Hz, PK2: 1.1 ± 0.16 Hz, Wilcoxon test, p=0.0411, n=19) (**Figures 3A, D**; **Supplementary Table 2**), whereas their amplitude presented no change (ctrl: -37 ± 2.5 pA, PK2: -37 ± 2.4 pA; **Supplementary Table 2**). Nevertheless, cluster analysis of the frequencies clearly showed that the recorded GnRH population was heterogenous, unresponsive (frequency: ctrl: 0.85 ± 0.11 Hz, PK2: 0.83 ± 0.10 Hz, n=11) and responsive (frequency: ctrl: 0.75 ± 0.15 Hz, PK2: 1.5 ± 0.33 Hz, n=8) groups existed (Mann-Whitney U-test, p=0.001) (**Figure 4**; **Supplementary Table 2**). Pretreatment of GnRH neurons with the prokineticin receptor blocker, PKRA7 eliminated the PK2-triggered elevation of the frequency of the mPSCs (PKRA7-ctrl: 0.75 ± 0.086 Hz, PK2: 0.70 ± 0.067 Hz, Student’s t-test, t= 1.065, df=9, p= 0.3145, n=10) (**Figures 3B, D**; **Supplementary Tables 2**, **3**). The highly selective PK2-receptor antagonist PC27 (1 µM) also prevented the effect of PK2 on mPSCs (PC27-ctrl: 0.72 ± 0.099 Hz, PK2: 0.68 ± 0.089 Hz, Student’s t-test, t= 1.679, df=9, p= 0.1275, n=10) (**Figures 3C, D**; **Supplementary Tables 2**, **3**). The finding is indicative of PK2 receptor expression in GnRH cells.

**Figure 5:**
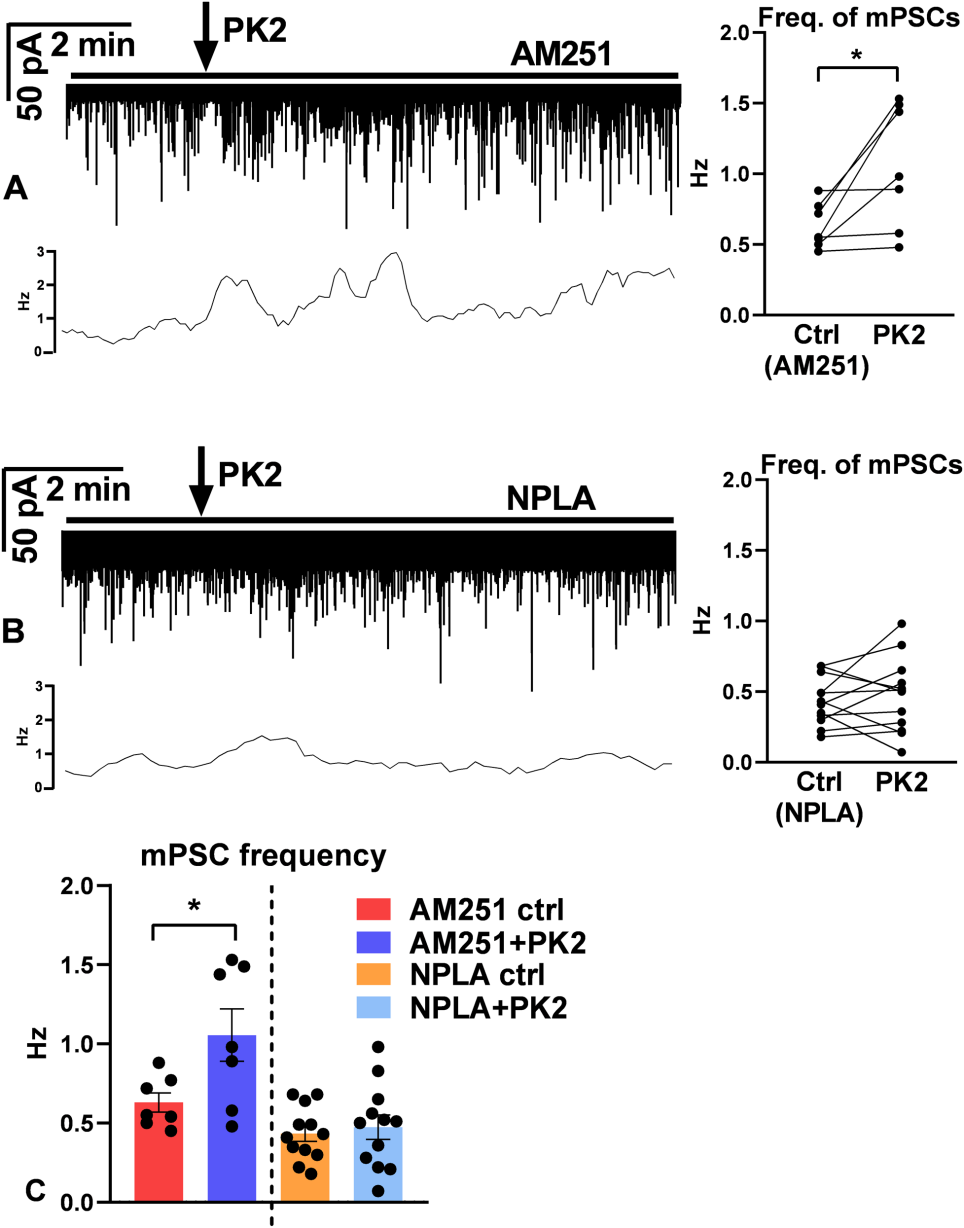
mPSC responses of GnRH neurons upon prokineticin 2 administration in the presence of AM251 or NPLA. **(A)** Prokineticin 2 (PK2) application (arrow) elevates frequency of mPSCs in GnRH neurons significantly in the presence of the inverse agonist of cannabinoid receptor 1 (CB1), AM251 showing that endocannabinoid retrograde mechanisms do not play role in the process. **(B)** In contrast, the neuronal nitric oxide synthase (NOS) inhibitor, NPLA prevented action of prokineticin 2. **(C)** Bar graphs confirm significant facilitatory effect of prokineticin 2 in the presence of AM251, but not in NPLA. Horizontal lines show presence of inhibitors. Frequency distribution curve below each recording shows changes in the frequency.

Changes in the frequency of mPSCs of GnRH neurons suggest involvement of retrograde endocannabinoid and/or NO signalling mechanisms (32). First, PK2 was applied in the extracellular presence of the cannabinoid receptor inverse agonist AM251 (1 µM). However, blockade of the retrograde endocannabinoid pathway didn’t eliminate the PK2-evoked increase in the frequency (AM251-ctrl: 0.63 ± 0.061 Hz, PK2: 1.1 ± 0.17 Hz, Wilcoxon test, p=0.0156, n=7) (**Figures 5A, C**; **Supplementary Tables 2**, **3**). In contrast, pretreatment of the slice with the nNOS-inhibitor NPLA (1 µM) prevented effect of PK2 (NPLA-ctrl: 0.43 ± 0.049 Hz, PK2: 0.47 ± 0.077 Hz, Student’s paired t-test, t=0.6338, df=11, p=0.5392, n=12) (**Figures 5B, C**; **Supplementary Table 2**).

### A subpopulation of GnRH neurons expresses PK2 receptor mRNA

**Figure 6:**
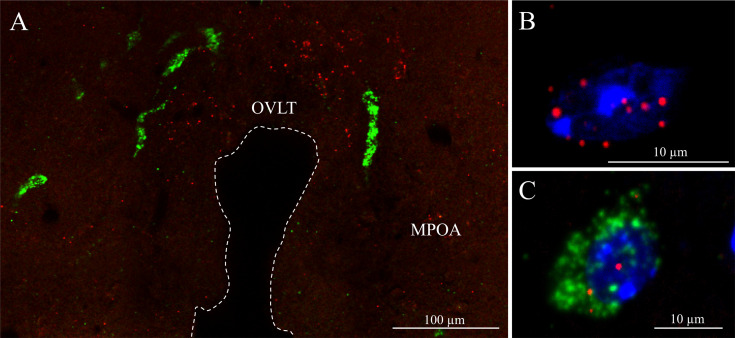
Simultaneous detection of GnRH neurons and PKR2-expressing cells by RNAscope hybridization technique in the basal forebrain **(A)** Distribution of GnRH mRNA (green)- and prokineticin receptor 2 mRNA (red)- expressing neurons in the medial preoptic area (MPOA) and the vascular organ of lamina terminals (OVLT). The optic recess of the third ventricle is indicated by dashed line. **(B, C)**. High power images of PKR2 mRNA expressing neurons with DAPI nuclear counterstaining (blue), enabling clear assignment of the signals to individual cells. The neuron depicted in B shows an intense PKR2 mRNA content. Co-expression of GnRH and PKR2 messages is presented in **(C)** Scale bar: 100 µm **(A)**, 10 µm **(B, C)**.

**Figure 7:**
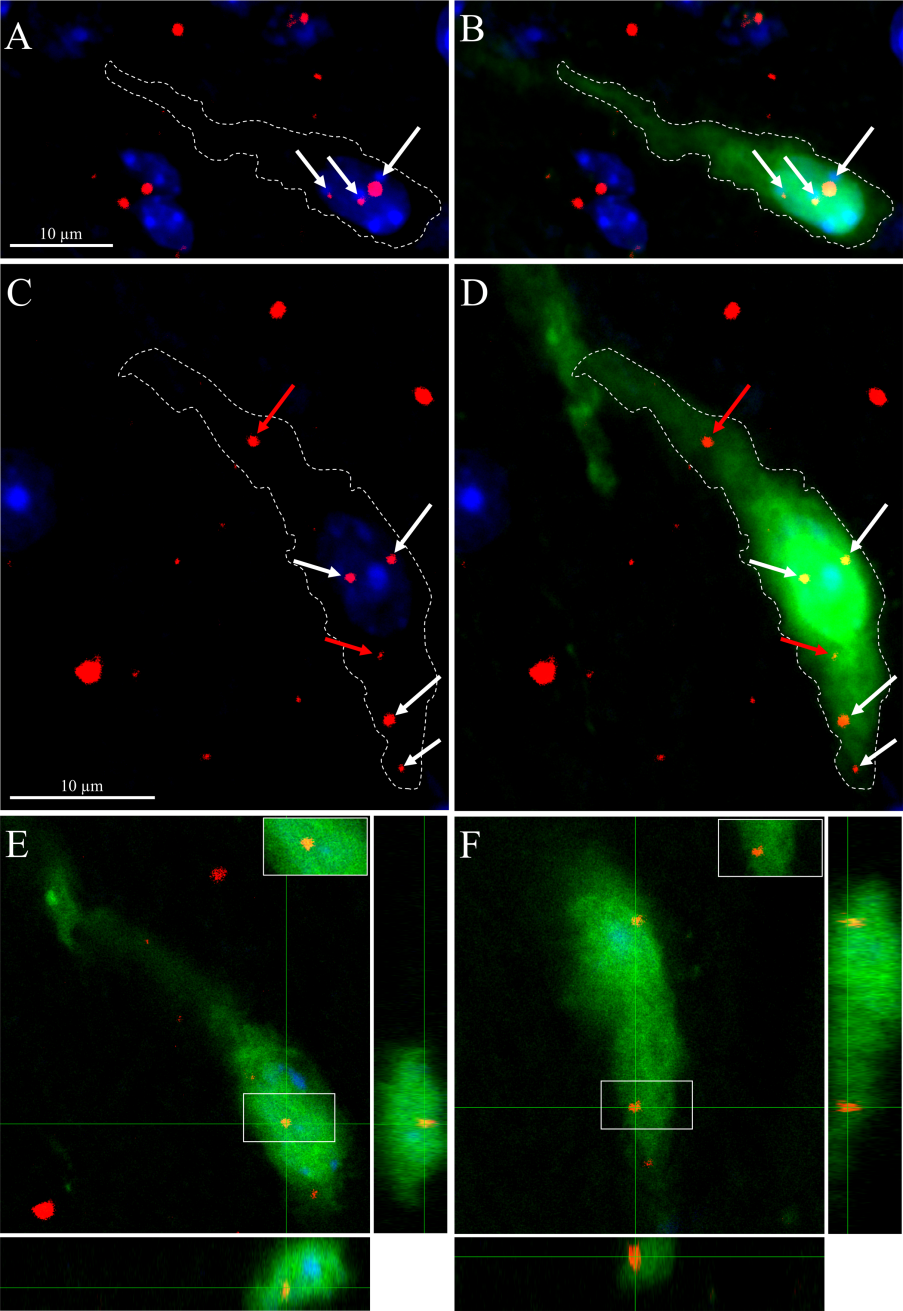
Occurrence and orthogonal analysis of PKR2 mRNA expression in GnRH-GFP neurons by RNAscope *in situ* hybridization. **(A)** Confocal optical slices (~20 × 0.3 µm) were merged to reconstruct the z-dimension of a section from the medial preoptic area (MPOA). Cell nuclei are labelled with DAPI (blue), while red puncta represent PKR2 mRNA signal (white arrows). White border outlines a GnRH (green) expressing neuron, shown in panel **(B-D)**. Further example of simultaneous localization of PKR2 and GnRH mRNAs in a fusiform neuron of the MPOA. Orthogonal analysis revealed that out of six PKR2 mRNA signals (arrows), only four were located within the GnRH neuron itself (white arrows), the rest (red arrows) appeared beneath the GnRH neuron. **(E, F)**. Orthogonal projections of two PKR2 mRNA signals (enframed areas and insets) from **(D)** confirm their localization within the reconstructed GnRH neuron. Scale bars: 10 µm.

The increased frequency of mPSCs evoked by PK2 in GnRH neurons in tetrodotoxin-treated slices indicates a direct regulatory action of PK2 on these cells, implying that they likely express PK2R. To address this issue, we examined the potential expression of PKR2 mRNA in GnRH-producing neurons using sensitive double-label RNAscope *in situ* hybridization. Simultaneous detection of PKR2 and GnRH mRNAs revealed that both transcripts occur in the territory of the diagonal band of Broca and the medial preoptic area (**Figure 6A**). The PKR2 message appeared in the perinuclear cytoplasm of the cell (**Figure 6B**). The high-resolution imaging showed co-expression of the hybridization signals at the level of single cells proving the expression of PKR2 mRNA in a subset GnRH neuron (**Figures 6C**, **7B, D**). To quantify the extent of PKR2–GnRH mRNA co-expression, confocal Z-stack images were analysed by examining each fluorescent channel (**Figures 6C**, **7A, C**) with orthogonal precision (**Figures 7E, F**).

In total, 4,358 optical slices (0.3-0.5 µm thickness) from 361 GnRH neurons in the septal–DBB–MPOA region were examined. Among these, 115 double-labelled neurons were identified, indicating that 31.9% (± 3.1% SD) of GnRH neurons expressed PKR2 mRNA (**Table 1**).

**Table 1:** Identification and quantification of PKR2 mRNA signal in GnRH neurons using RNAscope *in situ* hybridization technique.

Brain ID	Number of analysed GnRH neurons	Number of analysed optical sections	Number of GnRH neurons expressing PKR2 mRNA	Percentage of colocalization
1	82	1034	24	29.27
2	54	677	18	33.33
3	77	951	26	33.77
4	51	671	18	35.29
5	49	486	16	32.65
6	48	539	13	27.08
∑	361	4358	115	31.86

Thin (25 µm) slices of the basal forebrain from six female mice were prepared and subjected to RNAscope *in situ* hybridization to detect simultaneously PKR2 and GnRH mRNAs. The slices were then scanned using a confocal laser scanning microscope with a 60× oil-immersion objective. To precisely determine whether PKR2 mRNA signals are localized within GnRH neurons, optical sections of 0.3 µm thickness were acquired. In all, 4,358 optical slices from 361 GnRH cells were analysed. Based on this analysis and representative cell counting, approximately 31.9% of GnRH neurons were found to express PKR2 mRNA; the proportion varied moderately across samples (SD = 3.1%).

## Discussion

In the present study, we provide converging electrophysiological and molecular evidence that prokineticin 2 (PK2) regulates a subpopulation of gonadotropin-releasing hormone (GnRH) neurons in adult female mice. Specifically, (1) PK2 increased action potential firing, (2) enhanced miniature postsynaptic current (mPSC) frequency under tetrodotoxin (TTX) blockade, (3) produced effects that were prevented by prokineticin receptor 2 (PKR2) antagonists, (4) activated the retrograde nitric oxide (NO) signalling pathway and (5) its receptor was expressed in a subset of GnRH neurons identified using RNAscope *in situ* hybridization.

Application of PK2 significantly increased the firing rate of GnRH neurons; however, only 42.9% of the recorded neurons responded. Cluster analysis revealed two distinct groups of GnRH neurons - responders and non-responders - indicating functional heterogeneity within the population. In the responder group, PK2 exposure increased the firing frequency. Similar partial responsiveness to PK2 has been reported in other hypothalamic and circumventricular regions. In the hypothalamic paraventricular nucleus, PK2 increases the firing rate of both magnocellular and parvocellular neurons, partly through MAP-kinase–dependent mechanisms (41). Approximately 40% of neurons in the subfornical organ also exhibit depolarization and increased firing following PK2 exposure, attributable in part to modulation of sodium channels and a reduction in the delayed rectifier potassium current (42). In the area postrema, PK2 exerts mixed effects, inducing depolarization or hyperpolarization in distinct neuronal subsets (43). PK2 additionally enhances the firing of dorsally located neurons in the suprachiasmatic nucleus and modulates their activity through GABA_A_-receptor signalling (44). These findings of our study are consistent with earlier work showing that PKR2 antagonism transiently disrupts the oestrous cycle and lowers circulating luteinizing hormone (LH) concentrations (26), while chronic central PK2 delivery increases GnRH mRNA levels and stimulates gonadotropin and testosterone secretion (27). Together, these data support a role for PK2–PKR2 signalling pathway in the ongoing physiological modulation of the hypothalamic–pituitary–gonadal (HPG) axis.

PK2 may regulate GnRH neurons either directly or via upstream PKR2-expressing afferent systems. Several brain regions that express PKR2 (5) - including the septum, medial amygdala, medial preoptic area, suprachiasmatic nucleus, and arcuate nucleus - also send projections to GnRH neurons (45) and thus represent potential indirect regulatory pathways. Our intracellular blockade experiments, in which inhibition of G-protein–coupled receptor signalling prevented PK2-induced increases in firing, strongly support the existence of a direct PK2 regulatory channel of GnRH neurons.

To test this directly, we assessed synaptic events under TTX to eliminate action potential–dependent inputs. PK2 increased the frequency of mPSCs without affecting their amplitude in GnRH neurons, indicating modulation of presynaptic release probability. As in the firing rate analysis, only a subset of cells (42.9%) responded. PK2-induced increases in miniature excitatory postsynaptic currents have also been observed in magnocellular and parvocellular neurons of the paraventricular nucleus (38), suggesting that presynaptic facilitation is a shared feature of PK2 signalling in several neuroendocrine systems. The blockade of PK2-evoked changes in mPSC frequency by the PKR2 selective antagonist PC27 confirms the involvement of PKR2 in the regulation.

Prokineticin 2 (PK2) activated GnRH neurons through its specific receptor, PKR2. The physiological effect of PK2 was completely prevented by PKR2 antagonists and by pharmacological blockade of G-protein signalling. To elucidate the intracellular mechanisms involved, we investigated the signalling pathways downstream of PKR2 with particular emphasis on the potential involvement of retrograde signalling mechanisms. Previous work from our laboratory demonstrated that GnRH neurons generate retrograde endocannabinoid (2-arachidonoylglycerol; 2-AG) (33) and nitric oxide (NO) (32) signals that act on presynaptic GABAergic and glutamatergic terminals. Moreover, estradiol during proestrus (46), insulin-like growth factor-1 (IGF-1) (32), and secretin (47) were shown to activate GnRH neurons via retrograde NO signalling targeting their presynaptic GABAergic afferents. In the present study, blockade of the CB1 receptor with the inverse agonist AM251 did not alter the PK2-induced facilitation of mPSCs in GnRH neurons in the presence of tetrodotoxin (TTX). In contrast, inhibition of nitric oxide synthesis using N-propyl-L-arginine (NPLA) completely prevented the PK2-induced increase in mPSC frequency, indicating that NO signalling is essential for this effect. In addition to GnRH neurons, other neighbouring neurons might also release NO in response to PK2 exposure provided that they express PKR2 and capable of synthesizing NO.

Previous studies using conventional *in situ* hybridization (22) or transgenic reporter mouse model (5) did not detect PKR2 expression in mouse GnRH neurons. Using the more sensitive RNAscope technology, we found PKR2 mRNA expression in regions containing rodent GnRH neurons and uncovered that 31.9% of GnRH cells expressed PKR2 transcripts.

In theory, PK2 may utilize an alternative regulatory pathway to exert its effects on GnRH neurons. Because GnRH neurons express prokineticin receptor 1 (PKR1) transcripts (48) and PKR1 and PKR2 share approximately 85% sequence homology (6–8), it is plausible that PK2 could act through PKR1, provided that both receptors are expressed in these neurons. However, this putative mechanism has not been elucidated yet.

**Figure 8:**
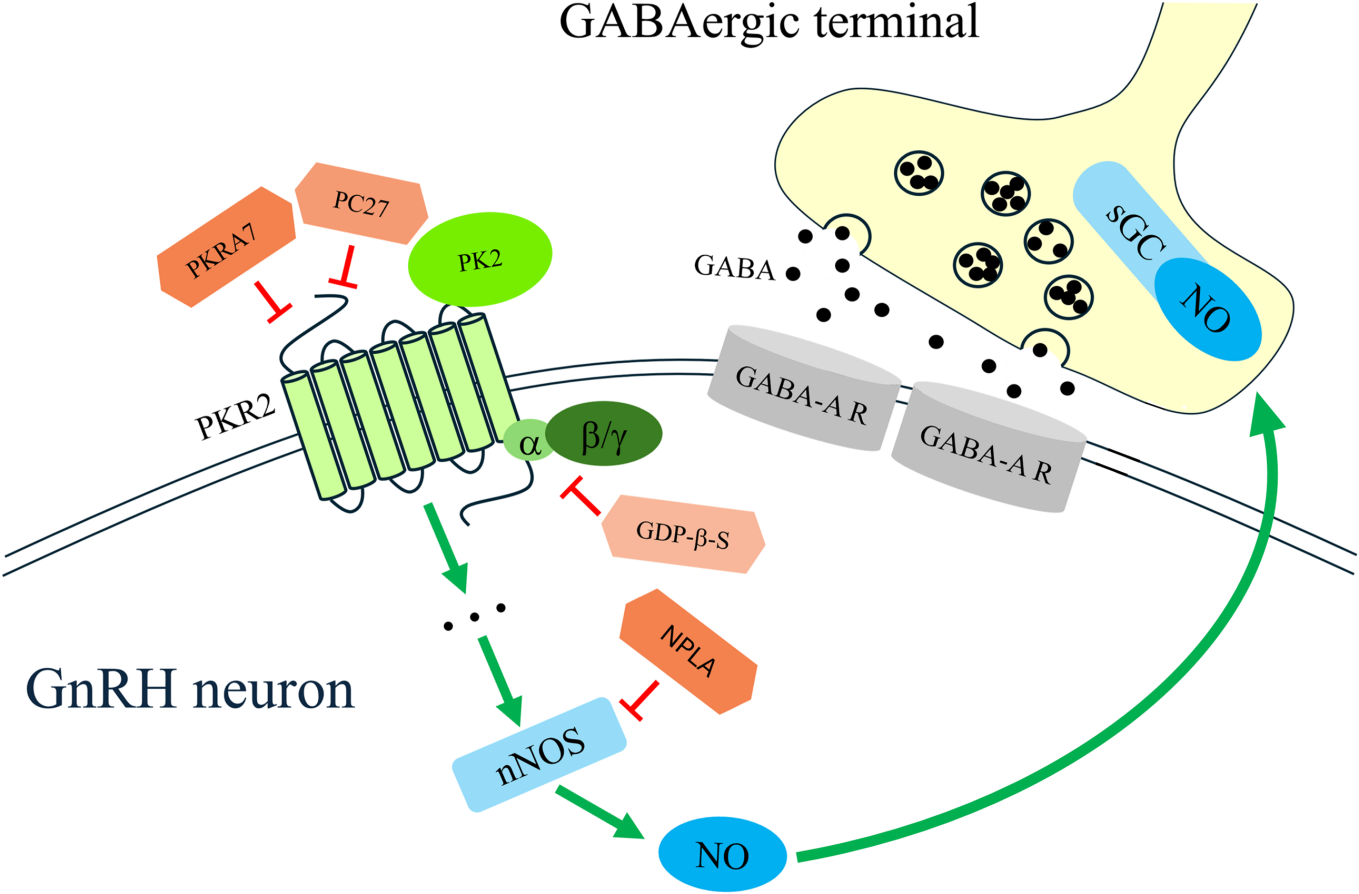
Schematic illustration of prokineticin 2 (PK2) signaling pathway in gonadotropin-releasing hormone (GnRH) neuron. PK2 activates PK2 receptor (PKR2), expressed in a subpopulation of GnRH neurons, thereby initiating an intracellular signaling cascade that leads to neuronal nitric oxide synthase (nNOS) activation and nitric oxide (NO) production. NO subsequently activates soluble guanylate cyclase (sGC) in presynaptic GABA terminals, enhancing the release of GABA, which in turn, increases the activity of GnRH cells. Theoretically, neighbouring neurons might also respond to PK2 and release NO, provided that they express PKR2 and nNOS.

In summary (**Figure 8**), our findings demonstrate that PK2 directly modulates the excitability and synaptic properties of a subpopulation of adult female GnRH neurons. Approximately one-third of these neurons express PKR2 mRNA and show electrophysiological responses to PK2. The results strengthen the evidence that PK2 contributes to the regulation of GnRH neuron activity and, by extension, plays an important role in the modulation of the HPG axis in adult female mice.

## Data Availability

The original contributions presented in the study are included in the article/**Supplementary Material**. Further inquiries can be directed to the corresponding author.
